# An internet of things labelled dataset for aquaponics fish pond water quality monitoring system

**DOI:** 10.1016/j.dib.2022.108400

**Published:** 2022-06-20

**Authors:** C.N. Udanor, N.I. Ossai, E.O. Nweke, B.O. Ogbuokiri, A.H. Eneh, C.H. Ugwuishiwu, S.O. Aneke, A.O. Ezuwgu, P.O. Ugwoke, Arua Christiana

**Affiliations:** a Department of Computer Science, University of Nigeria Nsukka, Nigeria; b Department of Environmental Biology and Zoology, University of Nigeria Nsukka, Nigeria; c ICT Unit, University of Nigeria Nsukka, Nigeria

**Keywords:** Aquaculture, Sensors, Microcontroller, Catfish, aquaponics, IoT

## Abstract

Aquaculture, which is the breeding of fishes in artificial ponds, seems to be gaining popularity among urban and sub-urban dwellers in Sub-Saharan Africa and Asia. Tenant aquaculture enables individuals irrespective of their profession to grow fishes locally in a little space. However, there are challenges facing aquaculture such as the availability of water, how to monitor and manage water quality, and more seriously, the problem of absence of dataset with which the farmer can use as a guide for fish breeding. Aquaponics is a system that combines conventional aquaculture with hydroponics (the method of growing plants in water i.e. soilless farming of crops). It uses these two technologies in a symbiotic combination in which the plant uses the waste from the fish as food while at the same time filtering the water for immediate re-use by the fish. This helps to solve the problem of frequent change of water. An Internet of Things (IoT) system consisting of an ESP-32 microcontroller which controls water quality sensors in aquaponics fish ponds was designed and developed for automatic data collection. The sensors include temperature, pH, dissolved oxygen, turbidity, ammonia and nitrate sensors. The IoT system reads water quality data and uploads the same to the cloud in real time. The dataset is visualized in the cloud and downloaded for the purposes of data analytics and decision-making. We present the dataset in this paper. The dataset will be very useful to the agriculture, aquaculture, data science and machine learning communities. The insights such dataset will provide when subjected to machine learning and data analytics will be very useful to fish farmers, informing them when to change the pond water, what stocking density to apply, provide knowledge about feed conversion ratios, and in predict the growth rate and patterns of their fishes.

## Specifications Table

**Table:** 

Subject	Aquaculture
Specific subject area	Aquaponics – Aquaculture (fish breeding) and Plant breeding (hydroponics)
Type of data	Table, Chart
How the data were acquired	The datasets were acquired using water quality sensors such as submersible temperature sensor, dissolved oxygen, turbidity, pH, ammonia, and nitrate sensors connected to ESP 32 [4], a 32-bit microcontroller. The microcontroller has an in-built WiFi module which enables the sensors’ data to be automatically uploaded to a cloud computing platform, known as Thingspeak IoT cloud, through an Internet of Things (IoT) network. C programming language was used to code the software program written to control the sensors with the Arduino 1.8.4 integrated development environment (IDE) known as sketch. The code was embedded in the microcontroller.The sensors used include:• 1. DF Robot pH sensor probe for Arduino version 2.0• 2. DF Robot Dissolved Oxygen sensor probe for Arduino• 3. DF Robot DC 5V TS-300B Turbidity Sensor Module Mixed Water Detection Module Water Quality Test Turbidity Transducer For Arduino• 4. Dallas DS18B20 temperature sensor.• 5. Ammonia detection sensor NH_3_ gas sensor module MQ137• 6. Nitrate detection sensor NO_3_ gas sensor module MQ135The dataset were cleaned, annotated and uploaded to the Kaggle Data Repository [13] for machine learning, which is licensed and free to download.
Data format	Raw (in .csv format)
Description of data collection	*The IoT system was programmed to automatically read six water quality parameters for each of the 12 fish ponds and transmit them to the cloud storage hosted by Thingspeak (*https://thingspeak.com/channels/1414062/*) every 5* s*. Besides the data collected by the IoT system, measurements were made fourth-nightly of the fish's weight and length by random sampling. About 10 to 15 samples were taken.*
Data source location	• Institution: University of Nigeria Nsukka• City/Town/Region: Nsukka/Nsukka/Enugu State• Country: Nigeria• Latitude and longitude (and GPS coordinates, if possible) for collected samples/data: Latitude: 6.85813, Longitude: 7.3968
Data accessibility	Repository name: KaggleDirect link to the dataset: https://www.kaggle.com/dataset/e81da8b7666dc7af41cdc3aa5ef96c5547e4f412598a030f40d444550965e34fData identification number (DOI): 10.34740/kaggle/dsv/2681778No access control is required to view or download the datasets.

## Value of the Data

• The sensor parameter readings from the fish pond gives realtime situation report on the condition of the water in the fish pond.
• Agricultural consultants, policy makers, and government agencies such as the ministry of agriculture will need the dataset to advise farmers and governments both for planning and predicting the performance of the fish.
• With the dataset being publicly available researchers, lecturers, and the machine learning community can use it to develop models that predict the growth rate of the fish, feed in-take and feed conversion rates.

## 1 Data Description

The data files are 12 comma separated values (.csv) format files, each representing an aquaponics fish pond as shown in Table 1. The files are named serially as in IoT_pond1.csv to IoT_pond12.csv

**Table 1:** Dataset table.

created_at	entry_id	Temperature(C)	Turbidity(NTU)	Dissolved Oxygen(mg/l)	pH	Ammonia (mg/l)	Nitrate(mg/l)	Length(cm)	Weight(g)

The table contains the date and time the data were collected, the entry id of each data from 1 to n. Then the next six columns contain the IoT water quality parameters. The last two columns were manually measured values as described above.

**Fig. 1:**
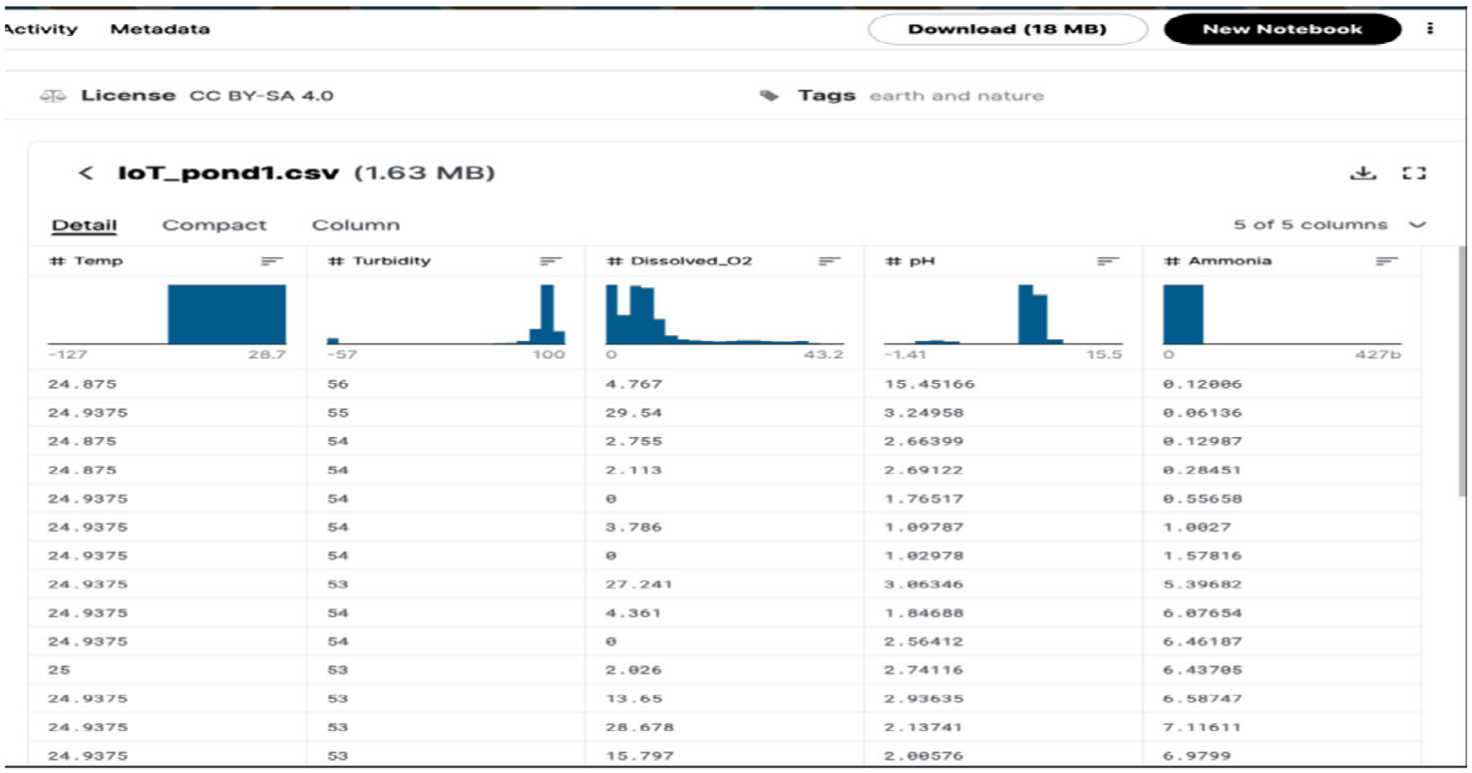
Screenshot of the pond1 aquaponics IoT datasets.

Fig. 1 shows a screen capture of the dataset on Kaggle website. Kaggle provides a graphical view of each of the dataset. The figure shows a section of the dataset as displayed on the Kaggle data repository. The columns in the figure represent the sensors such as the temperature, turbidity, dissolved oxygen, etc. The respective values of the sensors are also plotted in the figure using histograms.

## 2 Experimental Design, Materials and Methods

Water quality impacts on fish growth rate, feed consumption, and their general wellbeing [1,2]. Farmers’ ignorance of how to manage pond water has resulted to the death of fishes [3].

Six water quality sensors were used during the experiment for the data collection. These included temperature, turbidity, pH, dissolved oxygen, ammonia, and nitrate sensors. Each of the sensors was first of all calibrated according to industry and manufacturers’ specifications before being programmed and tested. The process of calibration involved using some calibration solutions, in some cases (such as sodium hydroxyl), as in the cases of the pH and dissolved oxygen sensors. The gas sensors were also calibrated with the relevant gases, after which the calibration codes were run. At the end of the modular programming, all the codes for the six sensors were integrated and the system was tested until the output was satisfactory.

The IoT system was programmed to automatically read the six water quality parameters for each of the 12 aquaponics fish ponds and transmit them to the cloud storage hosted by Thingspeak (https://thingspeak.com/channels/1414062/) every 5 s. Besides the data collected by the IoT system, measurements were made fourth-nightly of the fish's weight and length by random sampling. About 10 to 15 samples were taken from each pond, weighed on an electronic scale and measured with a measuring tape. The length and weight of the fish were then added as the 7th and 8th columns, respectively to the downloaded IoT water parameters tables for each of the 12 ponds. Thus, a complete dataset table was built having the IoT-generated water quality parameters and the manually measured length and weight parameters. IoT terminals may be incorporated with intelligence to take decisions, invoke actions and provide amazing services and improve quality of life [5,6].

*Experimental Setup*: The experiment was carried out in mobile aquaponics tarpaulin ponds measuring 2 × 1 × 1 m^3^. The ponds were used in an indoor production system. The volume of water was 1 × 2 × 0.9(m^3^). The ponds were filled at 0.9 m^3^ level of water and covered with a mosquito net. Seed beds of a chosen leguminous plant (amarantus spp.), the African spinach (Amaranthus hybridus), were constructed and fixed on top of the pond cover of the mobile tarpaulin pond. Each pond was connected to a 0.5 horsepower water pump for recirculation. Sensors of the different parameters were submerged into pond water following the methods of the pond controller system design described in sections above.

Physico-chemical parameters remain key factors in sustainable aquaculture production. These parameters include: Temperature, pH, dissolved oxygen, ammonia, turbidity, nitrate, and nitrite among others.

i. *Temperature***:** The Dallas DS18B20 waterproof temperature sensor was submerged in each of the 12 aquaponics ponds, being connected to the ESP 32 microcontroller. It senses the water temperature in degrees Celsius and returns the value in realtime to the microcontroller which in turns uploads the values to the cloud repository in the appropriate data table as shown in Table 1. The values were monitored to ensure they do not exceed the acceptable temperature for aquaculture species ranges between 25.5°C and 30.5°C.
ii. *pH***:** DF Robot pH sensor probe for Arduino version 2.0 was used to measure the degree of acidity or alkalinity in fish ponds to see if the values lie within the acceptable range of pH for tropical aquaculture is 6.5–8.2.
iii. *Dissolved Oxygen (DO)***:** DO has relatively lower solubility and availability in aquatic life than in terrestrial environments [7,8]. It is also required for several processes in the aquatic environment such as; oxidation, nitrification, and decomposition [9]. DF Robot Dissolved Oxygen sensor probe for Arduino is the sensor interfaced with the ESP 32 microcontroller for collecting the dissolved oxygen in the fish pond. The amount of dissolved oxygen in water is measured in milligrams per litre (mg/l).
iv. *Ammonia***:** Ammonia accumulates in fish ponds due to the breakdown of the protein rich fish feeds [10]. Ammonia detection sensor NH_3_ gas sensor module MQ137 was interfaced with the ESP 32 microcontroller. The Ammonia sensor was suspended above the pond water and senses the concentration and toxicity in ponds in realtime. The amount of ammonia is measured in milligrams per litre (mg/l).
v. *Turbidity***:** Turbidity measures the clarity or otherwise of a water sample. The turbidity unit is measured in NTU “Nephelometric Turbidity Units”. Turbidity sensors measure reflected (IR) light at 90 degrees to the source. The infrared phototransistor [11] will have a change in resistance itself and the change of voltage sensor will be obtained. This enables the DF Robot DC 5V TS-300B Turbidity Sensor Module Mixed Water Detection Module Water Quality Test Turbidity Transducer For Arduino connected to the ESP 32 microcontroller to measure the pond water turbidity.
vi. *Nitrate***:** is a by-product of nitrite oxidation during the latter stages of the nitrogen cycle and is present to some degree in all fish ponds [12]. Nitrate detection sensor NO_3_ gas sensor module MQ135 interfaced with the ESP 32 microcontroller was suspended above the pond water to measure the nitrate concentration in milligrams per litre (mg/l).

*Experimental Animals***:** 1000 numbers of five (5) week old fingerlings (mean initial weight: 2 ± 1.1 g) of the African catfish (*Clariasgariepinus*) were procured from a reputable hatchery and acclimatized for two weeks. During the acclimatization, the fish were fed with diets containing 42% crude protein at the rate of 7% of their body weights in divided rations of morning (8:00 h) and evening (4:00 h).

## Ethics Statements

The experiment complied with the ARRIVE guidelines and were carried out according to the UK Animals (Scientific Procedures) Act, 1986 and associated guidelines; EU Directive 2010/63/EU for animal experiments.

## Funding

*This work was supported by* the Meridian Institute under the Lacuna Fund for Agricultural Dataset 2020 [Grant No.: 0326-S-001, 2020], at 105 Village Place, Dillon, Colorado 80435, United States.

## CRediT authorship contribution statement

**C.N. Udanor:** Conceptualization, Methodology, Software, Supervision, Writing – original draft. **N.I. Ossai:** Methodology, Data curation. **E.O. Nweke:** Data curation, Writing – review & editing. **B.O. Ogbuokiri:** Software, Validation, Visualization, Data curation. **A.H. Eneh:** Software, Validation, Visualization, Data curation. **C.H. Ugwuishiwu:** Data curation. **S.O. Aneke:** Data curation. **A.O. Ezuwgu:** Data curation. **P.O. Ugwoke:** Data curation. **Arua Christiana:** Data curation.

## Declaration of Competing Interest

The authors declare that they have no known competing financial interests or personal relationships that could have appeared to influence the work reported in this paper.

The authors declare the following financial interests/personal relationships which may be considered as potential competing interests:

## Data Availability

Sensor Based Aquaponics Fish Pond Datasets (Original data) (Kaggle).
